# Identifying Candidate Gene–Disease Associations via Graph Neural Networks

**DOI:** 10.3390/e25060909

**Published:** 2023-06-07

**Authors:** Pietro Cinaglia, Mario Cannataro

**Affiliations:** 1 Department of Health Sciences, Magna Graecia University of Catanzaro, 88100 Catanzaro, Italy; 2 Data Analytics Research Center, Department of Medical and Surgical Sciences, Magna Graecia University of Catanzaro, 88100 Catanzaro, Italy

**Keywords:** graph neural network, gene disease associations, link prediction, neural network, deep learning

## Abstract

Real-world objects are usually defined in terms of their own relationships or connections. A graph (or network) naturally expresses this model though nodes and edges. In biology, depending on what the nodes and edges represent, we may classify several types of networks, gene–disease associations (GDAs) included. In this paper, we presented a solution based on a graph neural network (GNN) for the identification of candidate GDAs. We trained our model with an initial set of well-known and curated inter- and intra-relationships between genes and diseases. It was based on graph convolutions, making use of multiple convolutional layers and a point-wise non-linearity function following each layer. The embeddings were computed for the input network built on a set of GDAs to map each node into a vector of real numbers in a multidimensional space. Results showed an AUC of 95% for training, validation, and testing, that in the real case translated into a positive response for 93% of the Top-15 (highest dot product) candidate GDAs identified by our solution. The experimentation was conducted on the DisGeNET dataset, while the DiseaseGene Association Miner (DG-AssocMiner) dataset by Stanford’s BioSNAP was also processed for performance evaluation only.

## 1. Introduction

In recent years, a large amount of genomic and biological data has been studied in clinical research trials to correlate human diseases with genomics data, e.g., to define novel treatments. Disease profiling may include data from omics data (e.g., genomics, transcriptomics, and metabolomics) that could be related to disease susceptibility, progress and manifestation. The genes are crucial to understand the key factors involved in a correlated disease (e.g., molecular basis and biological mechanisms), as well as to evaluate the treatments and diagnosis. For instance, a phenotype may be caused by mechanisms which can be divided into several groups, where each one is characterized by a set of specific patterns and pathways that handle its activation. The knowledge related to this one could be very limited or misleading when the molecular mechanisms are unknown.

In bioinformatics, this topic has widely been tackled through the development of solutions for pattern recognition and data mining, besides data integration, in order to gather gene–disease associations (GDAs). A GDA is an association between a genetic variant with a disease or trait. The related branch of study focuses on investigating the divergences for one or more genes that could predispose to disease onset as well as be potentially responsible for the development of a specific disease phenotype. The GDAs are studied to identify the biological meaning for a given association that goes beyond pure randomness. To give an example, recent studies [1] proved the responsibility of aberrant interactions between mutant proteins, transcription factors and transcriptional co-activators that are the cause of the onset of Huntington’s disease. Authors based their own study on processing large genomic regions in a coordinated fashion to also evaluate disease progression and altered chromosomal clusters, according to these. Results support the notion of a common genome-wide mechanism of disruption of RNA transcription in the brain and periphery of Huntington’s disease patients. Similarly, computational models are also used to infer novel microRNA (miRNA)–disease associations by allowing the identification of promising miRNA–disease pairs for experimental validation [2,3,4,5].

Based on a network build by taking into account an exhaustive set of GDAs, the genotype of one or more polymorphisms will be seen more often in an individual carrying the trait, in relation to the probability that the latter is due to chance [6]. In this context, DisGeNET [7] integrates heterogeneous data sources to provide a set of curated and inferred GDAs. It identifies the diseases and the genes by using the Unified Medical Language System-Concept Unique Identifier (UMLS-CUI, or simply UMLS) and the Entrez Gene unique integer (i.e., gene identifier, or GeneID), respectively [8]. DisGeNET uses multiple data mining techniques on biological and literature data to build its own database [9].

The former was analyzed by integrating a set of existing databases (i.e., Universal Protein, Psychiatric Disorders Gene Association Network, Orphanet, Cancer Genome Interpreter, Comparative Toxicogenomics Database for Human, ClinGen, and Genomics England PanelApp), while the latter by performing the named entity recognition (NER) [10] of genetic variants on natural language texts retrieved both from the Single Nucleotide Polymorphism (SNP) database (i.e., dbSNP) [11] and the Universal Protein (UniProt) database [12]. Similarly, Stanford’s BioSNAP [13] provides a network-based dataset (Disease–Gene Association Miner, or DG-AssocMiner), containing a set of human GDAs. However, the latter contains much less information and GDAs than DisGeNET.

The real-world objects are usually defined in terms of their relationships or connections, which can be naturally expressed by using a graph (or network) model. In this paper, we will use the terms “graph” and “network” interchangeably.

In biology, depending on what the nodes and edges represent, we may classify several types of networks [14], such as gene regulatory networks, signaling networks, human disease networks, or protein interaction network. A network is also used to model gene–disease networks consisting of GDAs. In the last decade, the application of novel end-to-end deep learning (DL) paradigms boosted the research on pattern recognition and data mining in many domains [15,16,17]. For instance, the graph neural network (GNN) allows the modeling of graph data via NN, basing the computation on techniques originally designed for imaging. Briefly, an image can be represented by a matrix that can be transported into a Euclidean space to extract latent representation; therefore, the connections between the adjacent pixels can be treated as a graph. This approach may be considered biunivocal by adapting well-known paradigms from imaging to graph (or network) analysis [18]. We propose the following non-exhaustive example. Assuming G=(V,E) an undirected and unweighted graph, with *V* and *E*, two sets of *n* nodes and *m* edges, respectively. We can calculate a weight for each edge so that it is representative of the node’s neighborhood information. The resulting weighted graph $G^{\prime }$ can be used to compute a fixed length vector representation for each entity (i.e., embedding) based on the edge weights. Similarly to imaging, we could analyze a graph consisting of the nodes of interest (e.g., genes), instead of pixels.

As discussed, DL approaches for imaging base their own analysis on the Euclidean data obtained from the image data source. Therefore, we could apply the principle at the basis of a convolutional NN (CNN) for feature extraction, connectivity evaluation, as well as to exploit the pattern recognition [19,20].

Ultimately, a graph convolution can be generalized from a 2-dimensional (2D) convolution, and if an image is analyzed as a special case of a graph, then the latter may also be analyzed by taking into account its adjacent matrix; the only detail is to be able to relate the nodes of the graph through a system of weights that are representative of the characteristics of each node.

According to this statement, we modeled a GNN based on graph convolutions making use of multiple convolutional layers, and a point-wise non-linearity function following each layer; in detail, we used the rectified linear unit (*ReLU*) activation function. Fitting the input data source to the model required a preprocessing step to learn the weight matrix from its topology. The embeddings were computed for the input network built on a set of GDAs in order to map each node into a vector of real numbers in a multidimensional space, as well as to train our model. Subsequently, we used it to identify the existence of candidate links (i.e., GDAs) among the entities of the original network. The experimentation was conducted by using data in the DisGeNET database, while the DiseaseGene Association Miner (DG-AssocMiner) dataset by Stanford’s BioSNAP was also processed for performance evaluation only. Briefly, our contribution can be summarized as follows:

- Designing a GNN able to infer candidate GDAs by training an initial set of well-known and curated relationships between genes and diseases, modeled as a network;
- Embedding a biological network consisting of GDAs, based on its own topological features;
- Identifying candidate GDAs by exploiting link prediction via GNN.

The rest of the paper is organized as follows. Section 3 presents the design and implementation of our solution. Section 4 describes the model evaluation, and the explanatory tests for link prediction related to GDAs. Section 5 discusses the results. Finally, Section 6 concludes the paper.

## 2. Related Works

In this section, we report a set of solutions (i.e., methodologies, models, software tools and prototypes) based on GNNs. As introduced, GNNs represent a significant approach for graph-based studies. These may be applied to predict biological objects and/or their interaction in several fields, such as protein–protein interaction (PPI), gene interaction, drug–target interaction (DTI), and chemical stability prediction, as well as to exploit the topological structures in biological networks [21,22,23].

Wan et al. [24] proposed an inductive graph aggregator-based framework able to predict potential compound–protein interactions. The authors built a homogeneous graph starting from a compound–protein heterogeneous graph by integrating the ligand-based protein representations and overall similarity associations. They based the training process on low-dimensional node embeddings.

Similarly, Li et al. [25] developed a GNN model for diagnosis prediction. Authors retrieved the information for training on a graph built on temporal electronic health record data. A graph convolutional network (GCN) was used for the mentioned solutions.

In the past few years, GCNs have been widely used to develop several variants of GNNs [26]. They compute the input neurons with a set of weights (i.e., filters or kernels) that are exploited by using a sliding window to learn the features of interest for any neighboring nodes. This approach was originally designed for image analysis, in which a window slides across an image to acquire subsamples for object detection [27,28].

For instance, Li et al. [29] proposed a GCN for COVID-19 drug design. Authors based their own model on a multi-physical molecular graph representation by embedding various atom interactions in element-specific graph representations. They computed the embedding by only taking into account the distance-related node features, in order to overcome the issues related to feature extraction or generation. The model was trained by using data from several versions of the protein data bank binding (PDBbind) database.

A similar drug–side effects prediction was performed by Yu et al. [30] via a hybrid embedding GNN model, which integrates both graph-embedding and node-embedding modules. Therefore, the model was trained on a dual set of features, simultaneously.

Always resorting to using a GCN as a basis for their own model, Zhang et al. [31] proposed a *signed* GNN to predict deregulation types of miRNA–disease associations. The latter were processed as a signed bipartite network by taking into account both miRNA and disease nodes, as well as two types of edges representatives for a miRNA–disease association, in which the former was up- or down- regulated. The authors built their own model by learning the topological features of the input graph, through a node labeling computed on miRNA–miRNA functional similarity and disease–disease semantic similarity.

This issue has also been studied by Gao et al. [32]. Also in this case, the similarity was computed via node embeddings; however, the authors implemented a link prediction task to overcome the insufficient number of labeled similar disease pairs.

Kang et al. [33] applied a link prediction task to four biological network data (i.e., lncRNA–disease association, miRNA–disease association, protein–protein interaction, and drug–drug interaction), by including a GCN–encoder layer to improve the multi-link discrimination capabilities.

Finally, we investigated several issues addressed through the use of GNN in biological networks. One detail that is immediately apparent is that the mentioned solutions are based on GCNs. According to Zhang et al. [34], the reason could lie in the fact that GCN is the simplest model with fewer instructions and floating-point operations per second (FLOPS), which makes it the most commonly used architecture in real-life applications.

## 3. Materials and Methods

We designed a solution based on a GNN in order to identify candidate GDAs by training our model on the basis of an initial set of well-known and curated relationships among genes and diseases modeled as a network (i.e., DisGeNET). The model was built making use of convolutional layers, and an activation function based on *ReLU*; the latter is a point-wise non-linearity function following each layer. Furthermore, we mapped each node into a vector of real numbers in a multidimensional space by performing the node embeddings of the input network built on a set of GDAs. Each embedding is symptomatic for the features of the related node, obtained by evaluating the connections with its neighbors.

Node embeddings are used to train our model, and subsequently to predict the existence of candidate links (i.e., GDAs). In addition, fitting the original data source to the model requires a preprocessing step to learn the weight matrix based on the original graph topology.

Summarizing, we mapped the input graph into a lower-dimensional space (i.e., embedding) by using the model’s encoder, and a decoder to reconstruct the input graph from the embeddings via a dot product operator. Let us denote with *A* the adjacency matrix computed from the input graph, and with $A^{\prime }$ as the reconstructed adjacency matrix from the embedding. Our solution works by minimizing the loss function calculated on the difference between *A* and $A^{\prime }$.

### 3.1. Datasets

We briefly describe the specifications related to the datasets used by our solution for the testing and the performance evaluation. Their own data sources (i.e., DisGeNET and Stanford’s BioSNAP) are already presented in Section 1.

#### 3.1.1. DisGeNET (v7.0, Update 2020)

The last release of DisGeNET (v7.0, https://www.disgenet.org/dbinfo, accessed on 19 May 2023) contains 1,135,045 GDAs. It is provided as a SQLite database, which also includes variant–disease associations (VDAs) and disease–disease associations, as well as additional information about the biological objects involved within the associations (i.e., genes, diseases and variants).

The data are grouped into several sets (or versions), of which we used the “Curated” and “All” versions; the former refers to well-known GDAs related to humans (Homo sapiens), while the latter also includes inferred associations computed by integrating data derived from the text mining of the scientific literature and no-human repositories (e.g., MGI-Mouse Genome Informatics Database, and Rat Genome Database).

We report below the statistics for the two graphs modeled starting from the DisGeNET database.

*All*-version:

- Total nodes: 37,444;
- Gene nodes: 17,074;
- Disease nodes: 20,370;
- Edges: 1,122,238.

*Curated*-version:

- Total nodes: 20,924;
- Gene nodes: 9738;
- Disease nodes: 11,186;
- Edges: 168,992.

#### 3.1.2. DG-AssocMiner

In addition, we used a secondary dataset to evaluate our model on a different source. The latter is also related to the context concerning the GDAs. In detail, we retrieved the DG-AssocMiner provided by Stanford’s BioSNAP; this is available at https://snap.stanford.edu/biodata/datasets/10012/10012-DG-AssocMiner.html, accessed on 19 May 2023).

- Total nodes: 7813;
- Gene nodes: 7294;
- Disease nodes: 519;
- Edges: 21,357.

In this work, we strictly focused on DisGeNET, being composed of a larger dataset; we used DG-AssocMiner to provide a secondary feedback on the performance evaluated on the former. Results are reported in Section 4.

### 3.2. Preprocessing

We extracted all GDAs from DisGeNET by excluding additional information (e.g., aliases for genes and diseases, source used to identify an association, and other identifiers for external databases); the latter will be able to be integrated in post-processing, eventually.

Furthermore, we encoded the gene and disease identifiers in 32-bit floating point, according to the requirements of tensors (i.e., data structures used in linear algebra). Subsequently, we modeled an undirected, bipartite graph, where the nodes represent both genes and diseases, while the edges represent the GDAs. We repeated this step both for the “Curated” and “All” versions, in order to produce two independent graphs. The former was used for model training, validation and testing, while the latter as a reference for the identified GDAs.

We treated the GDAs as pairs (gene,disease). Let *G* be a set of genes and *D* be a set of diseases, such that $G=[g_{1},g_{2},\ldots ,g_{n}]$ and $D=[d_{1},d_{2},\ldots ,d_{m}]$, with *n* and *m*, respectively, the size of *G* and *D*. Formally, the cross-referencing may be computed to build the following domain: 

∀g∈G∃d∈D:f(g,d)→GDA

In the proposed solution, the pre-processing is performed automatically by a dedicated module which receives the following inputs: an edge list (i.e., GDAs), and two sets, from which to extract node information for gene and disease, respectively.

### 3.3. Graph Neural Network Architecture

We designed a GNN architecture based on graph convolution. The latter is a well-studied mathematical operator behind most GNN architectures [35]. As discussed before in Section 1 and Section 2, this approach considers the graph similar to an image, taking into account a generalization of this one, where each node is evaluated in reference to a set of adjacent neighbors, such as the pixels in an image. Therefore, the nodes in a specific layer are evaluated based on the neighbors’ features computed in the previous one, and these are transformed in a set of embedding vectors representing the related latent space.

We applied our model to link classification. Let us denote with G=(V,E) a given undirected graph, where $V=[v_{1},v_{2},v_{\ldots },v_{n}]$ is the set of nodes of size *n*, and $E=[e_{1},e_{2},e_{\ldots },e_{m}]$ the set of edges of size *m*. For a node $v_{i}$ (with 0≤i≤n), we can denote its features in the latent space as the vector $h_{i}$ such that the latter is computed by a function *f*; formally:

$$f:v_{i}\to h_{i}$$

Therefore, the whole set of neighbor’s features for $v_{i}$ can be represented through a tensor, where each row is the vector resulting from the adjacent neighbor’s feature. Formally, we can describe the function *g* for the evaluation of a link between two nodes $v_{i}$ and $v_{j}$ (with 0≤j≤n) as follows:

$$h_{i}=f\left(v_{i}\right)$$

$$h_{j}=f\left(v_{j}\right)$$

$$s_{ij}=fe\left(h_{i},h_{j},w_{ij}\right)$$

where $w_{ij}$ is the weight (normalized to [0−1] range) on the link between $v_{i}$ and $v_{j}$; note that $w_{ij}$ will be 1 for all edges, if the graph is unweighted.

We used *ReLU* as the activation function following each layer, and *Adam* [36] for the first-order gradient-based optimization of the stochastic objective function. Formally, *ReLU* can be defined as follows:

ReLU(x)=max(0,x)

Furthermore, we integrated an *encoder* within our model (i.e., *auto-encoder*), in order to compute the node embeddings by processing the input graph via multiple convolutional layers, based on the graph convolutional operator proposed by Kipf et al. [37].

We report the main specifications of the proposed model as follows:

- Layers: 2 convolutional layers;
- Activation function: *ReLU*;
- Dropout: p=0.5;
- Feature Size: 50;
- Decoder: *inner product decoder*.

Briefly, *dropout* refers to a simple way to prevent a NN from overfitting [38], by dropping out the nodes, both in the input and hidden layers; *p* is the probability of an element to be zeroed. The *dropout* randomly zeroes a set of elements within the input tensor; in detail, during the training step, it scales the outputs by a factor 1/(1−p), computing the identity function. According to Srivastava et al. [39], dropping a neuron with p=0.5 obtains the highest variance for the probability distribution of a NN architecture, for a wide variety of use cases (including ours).

### 3.4. Link (GDA) Prediction

The proposed solution uses a *Decoder*, to perform the link predictions via binary classification. It needs a set of negative links that are randomly included into the validation and testing sets; these are excluded from the training set to evaluate only the original graph structure.

The link prediction is performed by computing the dot product (or scalar product) of the node embeddings for all pair of nodes, and by evaluating the probability of edge existence as the resulting score from the aggregation of all embeddings for each pair of nodes. Therefore, a candidate link between a pair of nodes $\left(v_{i},v_{j}\right)$ is evaluated in reference to the calculated dot product between their respective embeddings. We calculated the dot products via the Einstein summation convention on the operands, for tensor contractions.

Resulting candidate links can be discriminated by applying a threshold, or by extracting a defined number among those with the highest value. Since a threshold is not defined in the literature, we opted for an empirical evaluation.

**Figure 1:**
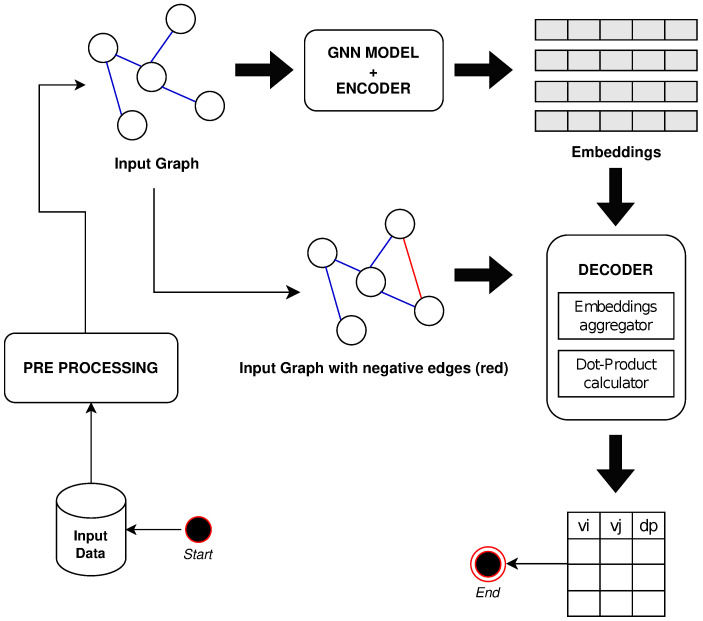
A non-exhaustive workflow of the proposed solution is reported.

Figure 1 shows a non-exhaustive workflow of the proposed solution.

### 3.5. Evaluation Criteria

We assessed the overall effectiveness of the proposed model, by employing two well-known key performance indicators (KPI): average precision (AP), and area under the receiver operating characteristic (ROC) curve (i.e., AUC or AUROC) [40].

The AP represents a precision–recall curve through a weighted average value of precision. It is defined as follows:

$$AP=\sum_{n}^{}\left(Recall_{n}-Recall_{n-1}\right)\times Precision_{n}$$

$$Precision=\frac{True\;Positives}{True\;Positives\;+\;False\;Positives},\;\mathrm{and}$$,

$$Recall=\frac{True\;Positives}{True\;Positives\;+\;False\;Negatives}$$

The ROC curve shows the relationship between sensitivity and specificity, for every possible cut-off. It takes into account the true positive rate (TPR) and false positive rate (FPR). It is possible to detect a unique threshold value for each local maximum in the difference curve. It will be the sensitivity parameter useful to measure how many flat points we should expect before producing a knee/elbow, into the plot [41,42,43]. A larger sensitivity value will detect a more conservative threshold. We evaluated the best threshold (or Best, in the figures) via Youden’s index (or *J*). It will be the maximum value of *J*, among all ones calculated for each point of the ROC curve [44]. Formally, *J* is defined as follows:

J=sensitivity+specificity−1

with sensitivity=truepositives/(truepositives+falsenegatives), and specificity=truenegatives/(truenegatives+falsepositives).

Of significant interest is the evaluation of the AUC computed on the ROC curve. It is a performance measurement used to evaluate classification models with different settings. Therefore, we used it to evaluate the model in terms of accuracy. Formerly, it is defined as follows:

AUC=∑TPRΔFPR; where

$$TPR=\frac{\left|True\;Positives\right|}{\left|True\;Positives\;\cup \;False\;Negatives\right|},\;\mathrm{and}$$

$$FPR=\frac{\left|False\;Positives\right|}{\left|False\;Positives\;\cup \;True\;Negatives\right|}$$

### 3.6. Implementation and Deployment

We implemented the proposed solution in Python (version 3), by using the Graph Auto-Encoder (GAE), belonging to the PyTorch framework [45]. GAE is an end-to-end trainable neural network model for unsupervised learning on graph-structured data.

Table 1 reports a list of the main PyTorch’s modules used in the implementation of the proposed solution.

Note that our implementation is optimized to be executed on a graphics processing unit (GPU).

We deployed our solution on Google Colaboratory (Colab; https://colab.research.google.com, accessed on 19 May 2023). To date, Colab (free plan) allows connecting to a session with the following specifications:

- CPU: Intel Xeon CPU @2.20 GHz;
- Memory: 12 GB;
- GPU: Tesla K80.

Note that Colab (free plan) could limit the session according to both the time of use and the workload of the server hosting it.

## 4. Results

We tested our solution by using the DisGeNET database as source, using both the *Curated-* and *All-*versions. The former was used to train and to evaluate the model in terms of AUC and AP, as well as to identify a set of candidate GDAs in a real use case (i.e., link prediction). The latter was used to evaluate our predictions by using no-human resources. We removed from the *All-*version the GDAs included in the *Curated-*version, in order to eliminate redundancy. Furthermore, we investigated scientific literature to evaluate a probable truthfulness (or hypothetical match) from recent studies, for the candidate GDAs not available in the *All-*version of DisGeNET.

Finally, we applied the proposed solution on a secondary dataset, to evaluate the model performance on a different data source as well as to corroborate the results obtained by processing the DisGeNET datasets.

### 4.1. Model Performance

**Figure 2:**
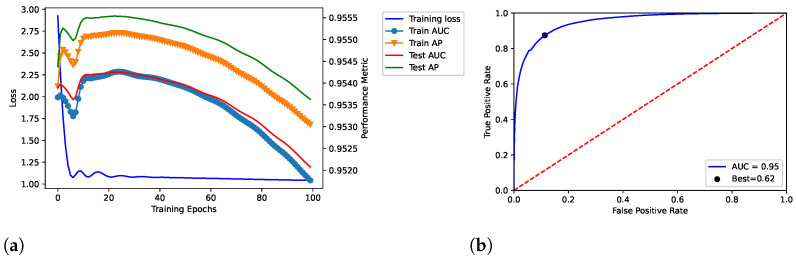
Dataset: DisGeNET. Training, validation and testing throughout 100 epochs; dataset: *Curated-*version. (**a**) Loss curve across the training process over the epochs. In addition, TPR (Sensitivity) and FPR (1−Specificity) were related through the ROC curve in (**b**) by also reporting the related AUC and best threshold (i.e., “Best”).

**Figure 3:**
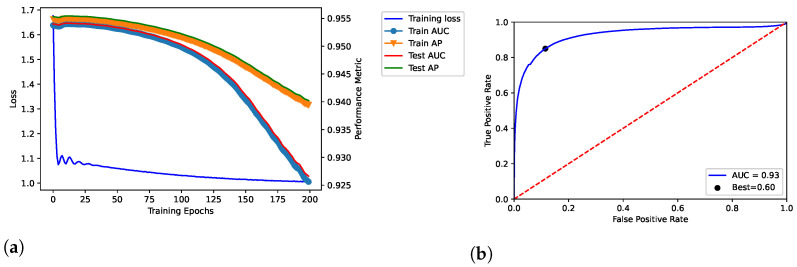
Dataset: DisGeNET. Training, validation and testing throughout 200 epochs; dataset: *Curated-*version. (**a**) Loss curve across the training process over the epochs. In addition, TPR and FPR were related through the ROC curve in (**b**), by also reporting the related AUC and best threshold (i.e., “Best”).

**Figure 4:**
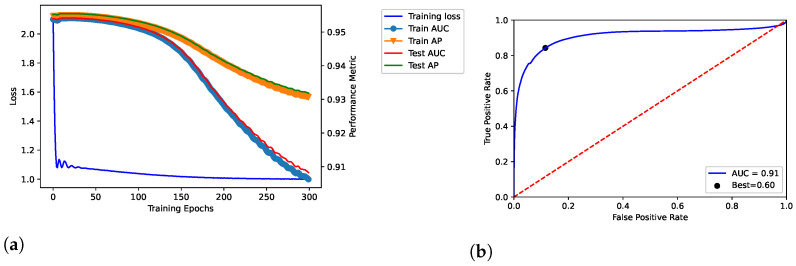
Dataset: DisGeNET. Training, validation and testing throughout 300 epochs; dataset: *Curated-*version. (**a**) shows the loss curve across the training process over the epochs. In addition, TPR and FPR were related through the ROC curve in (**b**), by also reporting the related AUC and best threshold (i.e., “Best”).

We trained the proposed model throughout 100, 200, and 300 epochs, by showing the results in Figure 2, Figure 3 and Figure 4, respectively. In addition, we evaluated empirically the optimal number of features by performing the training, validation and testing throughout 100 epochs (see Table 2); as shown, it obtained the best result in terms of AUC.

Based on the same specification and configuration, we also tested our solution on DG-AssocMiner to evaluate the performance in a different dataset and to corroborate our results. The resulting AUC and best threshold is shown in Table 3.

We performed an independent samples *t*-test, to detect mean differences in “Best Threshold” between the two presented datasets. The two-tailed *p*-value equals 0.0106; by conventional criteria (i.e., *p*-value < 0.05) it can be considered statistically significant. The AUC between the two datasets is the same for all experiments; therefore, we cannot analyze perfect data with a *t*-test, and the statistical test on this parameter was omitted.

### 4.2. Identification of Candidate GDAs

Firstly, we studied the *Curated-*version of DisGeNET by performing a node-level descriptive statistics of the resulting network; Table 4 shows the 10 genes and 10 diseases with the highest degree. These should be among the most recurring in the next test.

Subsequently, we applied our solution to the described network, to identify a set of candidate GDAs. The top-15 ones (highest dot product) are reported in Table 5. In addition, we correlated these with the GDAs inferred by DisGeNET in its own *All-*version, by integrating no-human datasets. We removed from the *All-*version the GDAs included in the *Curated-*version in order to eliminate redundancy.

Where a match with DisGeNET *All-*version was not found, we reported the PubMed IDentifier (PMID) of the articles, which may corroborate the candidate GDA of interest. In other words, for each predicted GDA not present in DisGeNET, we manually investigated the literature to find a scientific article that reports biological evidence of that GDA.

## 5. Discussion

The model related to the proposed solution reported very satisfactory performance. According to the loss curves (training loss, train AUC, train AP, test AUC, and test AP) as reported in Figure 2a, Figure 3a, and Figure 4a, the proposed model was able to identify positive links with high sensibility, without interference from the negative links and by reporting a low number of false positives. The related values of AUC are reported in Figure 2b, Figure 3b, and Figure 4b.

In our tests, AUC values demonstrate what was observed. The average value of AUC is 0.93, and it refers to a number of correct predictions equal to 93% of the total ones. In addition, the results report an AUC of 0.95 for the number of epochs (100) our model was ultimately trained on; the same experiments replicated on the DS-AssocMiner dataset produced a performance evaluation of 0.95 in terms of AUC. Therefore, 95% of predictions can be considered correct for the final model, being that the ones obtained for 200 and 300 epochs were discarded.

Summarizing, the proposed solution reported an excellent value of AUC in all tests. According to Nahm et al. [46], the interpretation of AUC may be addressed as follows:

- AUC≥0.9: Excellent;
- 0.8≤AUC<0.9: Good;
- 0.7≤AUC<0.8: Fair;
- 0.6≤AUC<0.7: Poor;
- 0.5≤AUC<0.6: Fail;
- AUC<0.5: Incorrect.

It is possible to note that once the 100 epochs are exceeded, the model begins to show overfitting. Furthermore, the empirical evaluation of the optimal number of features reports 50 as the optimal value to obtain the best results in terms of AUC and best threshold. As discussed, in the final model, we defined 100 and 50 as values for the number of epochs and features, respectively.

In addition, we tested a real use case, proceeding to identify the Top-15 candidate GDAs (highest dot product) inferred by the proposed solution (Table 5). We evaluated an existing or probable relationship between these and what was inferred by the DisGeNET (*All-*version purged of redundancies with the *Curated-* one). We also investigated the scientific literature by using the Pubmed search engine (https://pubmed.ncbi.nlm.nih.gov/, accessed on 19 May 2023), provided by the National Center for Biotechnology Information (NCBI), by reporting in Table 5 the Pubmed ID (PMID) of the articles that corroborate the possible correlations. Note that the PMID is reported only where a match with DisGeNET *All-*version was not found since the latter is already validated.

According to Table 5, we found a 47% (7/15) match between the GDAs already validated by the DisGeNET *All-*version, and the candidate ones predicted by our solution. Of the remaining 8 candidate GDAs, an existing or probable relationship was found for 7/8 of these by studying the scientific literature. Definitely, 14/15 candidate GDAs, 93.3% (14/15), had a positive match.

Deepening the study of our Top-15 candidate GDAs, we can see that the associations are referable to the following genes and diseases:

- Genes: TNF, SOD2, POMC, IL6, and TP53.
- Diseases UMLS (and Description): COO40517 (Gilles de la Tourette syndrome), C0006413 (Burkitt Lymphoma), C0079588 (Ichthyosis, X-Linked), and C0009404 (Colorectal Neoplasms).

For instance, *TNF* is a protein coding gene, which encodes a multifunctional proinflammatory cytokine, belonging to the tumor necrosis factor (i.e., *TNF*) super-family. The DisGeNET *All-*version reports 2/3 associations inferred by our solution; in detail, it reports an explicit association with *C0006413* and *C0009404*. Furthermore, the association with *C0009404* could be inferred from *PMID:25256363* [47], where Keszler et al. investigated two TNF promoter polymorphisms on the genetic susceptibility to Tourette syndrome (TS); the authors concluded by reporting: “Based on these findings, the *TNF* -308 G-allele can be associated with Tourette syndrome”. Similarly, the mentioned genes have been studied in the literature to evaluate the candidate associations inferred by our solution, but have not yet been included in DisGeNET.

Furthermore, we showed a greater recurrence of the nodes having a high-level degree (Table 4) among our candidate GDAs. This result was expected, in that the GCNs, as well as the GNNs making use of convolutional layers, express the accuracy by also taking into account the node’s degree. Indeed, from empirical observations, a GNN embeds the features with more accuracy during the training and testing, for nodes with the highest degree, even if these are under-represented [48].

Summarizing, the results showed an AUC of 95% for training, validation and testing throughout 100 epochs, which, in the real case, translated into a positive response for 93% of the candidate GDAs predicted by our solution.

## 6. Conclusions

GNN allows using a NN for graph data modeling, with relevant advantages in the context of node classification, link prediction and graph classification. In this paper, we presented a solution for GDAs prediction based on GNN. Our model was based on graph convolutions and node embeddings. The latter were computed for the input network built on a set of GDAs, in order to map each node into a vector of real numbers in a multidimensional space. Our tests were conducted by using the DisGeNET *Curated-*version for model training, while we used the *All-*version in conjunction with the literature to evaluate the candidate GDAs identified in a real use case. Results show an excellent AUC (95% throughout 100 epochs) for training, validation and testing throughout 100 epochs, which in the real case translated into a positive response for 93% of the Top-15 (highest dot product) candidate GDAs identified by our solution. Briefly, our results may be summarized as follows: we found a 47% match (7/15) between the GDAs already reported in the literature by the DisGeNET *All-*version, and the candidate ones identified by our solution; of the remaining 8, an existing or probable relationship was found for 7/8 of these by studying the scientific literature.

Future directions related to GDA are mainly focused on inferring novel associations, as well as how to handle (e.g., store and visualize) the relevant data both in terms of accessibility and scalability. The study of GDAs has an important impact on investigating novel associations between genes (or variants) and diseases. It provides a relevant effort for improving the knowledge for disease etiology [49], being an area of active research, for which in-depth studies are essential, particularly focused on the validation of the predictions made by bioinformatics methodologies, e.g., based on machine learning and deep learning.

## Figures and Tables

**Table 1:** Main PyTorch’s modules used in the implementation of the proposed solution.

Name	Description	Documentation
PyTorch Geometric (PyG)	It consists of several methods for geometric deep learning.	https://pytorch-geometric.readthedocs.io (accessed on 19 May 2023)
Sparse	It allows optimized sparse matrix operations on tensor.	https://pytorch.org/docs/stable/sparse.html (accessed on 19 May 2023)
NN	It provides several NN layers (e.g., convolutional layers).	https://pytorch.org/docs/stable/nn.html (accessed on 19 May 2023)
Tensor	It allows handling tensors.	https://pytorch.org/docs/stable/tensors.html (accessed on 19 May 2023)

**Table 2:** Dataset: DisGeNET. Training, validation and testing throughout 100 epochs, by incremental number of features. Bold font indicates the best result.

No. of Features	AUC	Best Threshold
25	0.95	0.62
**50**	**0.95**	**0.63**
75	0.95	0.62
100	0.95	0.61

**Table 3:** Dataset: DG-AssocMiner. Training, validation and testing throughout 100 epochs, by incremental number of features. Bold font indicates the best result.

No. of Features	AUC	Best Threshold
25	0.95	0.60
**50**	**0.95**	**0.62**
75	0.95	0.60
100	0.95	0.61

**Table 4:** Node-level descriptive statistics for the network built from DisGeNET (*Curated-*version). We reported the 10 genes and 10 diseases with the highest degree.

Gene Name	Node Degree	Disease UMLS	Disease Description	Node’s Degree
TNF	340	C0006142	Malignant neoplasm of breast	1074
SOD2	285	C0036341	Schizophrenia	1031
IL6	270	C0023893	Liver Cirrhosis, Experimental	774
POMC	241	C0009402	Colorectal Carcinoma	702
PTGS2	239	C0376358	Malignant neoplasm of prostate	616
TP53	232	C0033578	Prostatic Neoplasms	616
IL1B	231	C0678222	Breast Carcinoma	538
MTHFR	192	C0005586	Bipolar Disorder	536
NOS2	184	C1458155	Mammary Neoplasms	527
PTEN	182	C4704874	Mammary Carcinoma, Human	525

**Table 5:** Top-15 candidate GDAs (highest dot product) predicted by our solution, of which 47% (7/15) matched DisGeNET (*All-*version). Where a match with DisGeNET *All-*version was not found, the PMID of an article highlighting a possible match was found; we marked a GDA as *untraceable* when no match was found.

Gene	Disease		Reference
	UML	Description	
TNF	C0040517	Gilles de la Tourette syndrome	PMID: 25256363
TNF	C0006413	Burkitt Lymphoma	**DisGeNET (All)**
TNF	C0079588	Ichthyosis, X-Linked	**DisGeNET (All)**
SOD2	C0040517	Gilles de la Tourette syndrome	PMID: 31468582 (SOD, enzyme)
SOD2	C0006413	Burkitt Lymphoma	PMID: 28483518
SOD2	C0079588	Ichthyosis, X-Linked	PMID: 28540003 (*SOD1)
TNF	C0009404	Colorectal Neoplasms	**DisGeNET (All)**
POMC	C0040517	Gilles de la Tourette syndrome	*untraceable*
POMC	C0006413	Burkitt Lymphoma	PMID: 29296973
SOD2	C0009404	Colorectal Neoplasms	**DisGeNET (All)**
IL6	C0040517	Gilles de la Tourette syndrome	PMID: 35087475
IL6	C0006413	Burkitt Lymphoma	**DisGeNET (All)**
TP53	C0040517	Gilles de la Tourette syndrome	**DisGeNET (All)**
POMC	C0079588	Ichthyosis, X-Linked	PMID: 22289416
TP53	C0006413	Burkitt Lymphoma	**DisGeNET (All)**

## Data Availability

Gene–Disease Associations used for training and testing our model during the current study are available on DisGeGNET database (https://www.disgenet.org, accessed on 19 May 2023). Results are available on our own GitHub Repository (https://github.com/pietrocinaglia/gnn_gda, accessed on 19 May 2023).
